# Neuroinflammation and Neuromodulation in Neurological Diseases

**DOI:** 10.3390/bs9090099

**Published:** 2019-09-12

**Authors:** Maria de los Angeles Robinson-Agramonte, Carlos-Alberto Gonçalves, Roberto Farina de Almeida, Alina González Quevedo, Sandra Chow, Luis Velázquez Pérez, Amado Díaz de la Fé, Patricia Sesterheim, Diogo Onofre Gomes Souza

**Affiliations:** 1 Cuban Society of Neuroimmunology, Havana 11300, Cuba; 2 International Center for Neurological Restoration, Ave 25 # 15805 b/w 158 and 160, Havana 11300, Cuba; 3 Dept of Biochemistry, Federal University of Rio Grande do Sul, Porto Alegre 90035-003, Brazil; 4 Universidade Federal de Ouro Preto, OP 3540-00, Brazil; 5 National Institute of Neurology and Neurosurgery, Havana 10400, Cuba; 6 MSL N&I Centro America y el Caribe.Biopharm Medical Affairs, Guatemala; 7 Center for the Research and Rehabilitation of Hereditary Ataxias (CIRAH), Holguin 80100, Cuba; 8 Institute of Cardiology of Rio Grande do Sul, Experimental Center, Porto Alegre 90650-090, Brazil

**Keywords:** neuroimmunology, neurodevelopmental disorders, Strock neurodegenerative disorders, non-invasive brain stimulation, Demyelinating disease, SCA2 Cerebellar Ataxy, neuroimmunomodulation

## Abstract

Neuroimmunology is a relatively young science. This discipline has emerged today from the research field as a mature and fully developed innovative research area that integrates not only pure topics of neuroimmunology, but also expands on wider fields such as neuroplasticity, neuronal reserve and neuromodulation in association with clinical events, amongst which behavioral disorders stand out. The Cuban School of Neuroimmunology—a recent meeting that took place in Havana, Cuba—focused on topics based on the molecular mechanisms of neuroinflammation in neurological disorders involving behavioral manifestations, such as multiple sclerosis (MS), autism, cerebellar ataxias, Alzheimer´s disease and stroke among others, as well as on the use of new interventional technologies in neurology. Professor Luis Velazquez, from the Cuban Academy of Sciences, dictated an interesting lecture on Spinocerebellar ataxias, a genetic disorder where recent hypotheses related to the influence of neuroinflammation as a neurobiological factor influencing the progression of this disease have emerged. At the same time, the use of new interventional technologies in neurology was discussed, including those referring to novel disease modifying therapies in the course of MS and the use of transcranial magnetic stimulation in several neurological diseases, the latter reinforcing how interventional strategies in the form of non-invasive bran stimulation can contribute to physical rehabilitation in neurology. This paper summarizes the highlights of the most relevant topics presented during the First Cuban School of Neuroimmunology, organized by the Cuban Network of Neuroimmunology, held in June 2019.

## 1. Introduction

Neuroimmunology is a relatively young science. This discipline has emerged today from the research field as a mature and fully developed innovative research area that integrates not only pure topics of neuroimmunology, but also expands on wider fields such as neuroplasticity, neuronal reserve and neuromodulation. Several diseases were revised in interesting lectures to offer a major understanding of the molecular processes underlying neuroimmune pathology in neurodegenerative, neurodevelopmental and neurovascular diseases, as well as to understand some vulnerabilities occurring in these disorders [1,2,3,4,5].

From this point of view, several diseases were revised in interesting lectures to offer a greater understanding of the molecular processes underlying neuroimmune pathology in neurodegenerative, neurodevelopmental and neurovascular diseases, as well as, to understand some comorbidities that affect disease evolution and outcome [1,2,3,4,5]. In this context, the lecture on Spinocerebellar Ataxias type 2 presented by Professor Luis Velazquez from the Cuban Academy of Sciences, showed how molecular processes underlying neuroinflammation can also affect genetic disorders influencing onset and disease progression [1,2,3,4,5].

## 2. Lecture Highlights

Astrocytes as Integrative Elements in the Neuroinflammation Associated with Neurodegenerative Diseases

Carlos-Alberto Gonçalves and Elena Noris García. E mail:casg@ufrgs.br 

**Figure 1:**
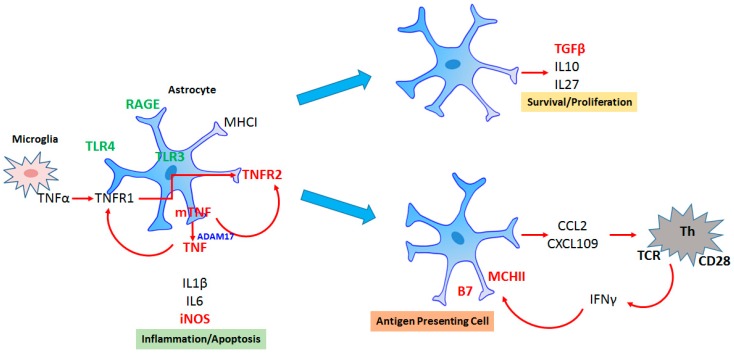
Heterogeneity of reactive astrocytes during inflammation. Astrocytes respond directly to inflammatory stimulus (e.g., via TLR4: toll like receptor 4 or indirectly to inflammatory cytokines by cytokine receptors. In basal conditions, they secrete small quantities of cytokine TNF (tumor necrosis factor) and express only TNFR1 (tumor necrosis factor receptor 1). When exposed to inflammation, TNF production is increased, as well as the expression of TNFR2, which is commonly expressed by immune cells. TNFR2-astrocytes can generate both anti-inflammatory astrocytes able to express TGF (tumor growth factor) and IL-10 and pro-inflammatory astrocytes which are able to release chemokines and attract T lymphocytes, which, in turn, release interferon gamma (IFNγ), inducing the expression of MCH class II in astrocytes, and transforming them into antigen-presenting cells.

Astrocytes are the most abundant glial cell in the brain tissue and, functionally, are equally, or more, heterogeneous than neurons [6]. They modulate synaptic communication, affecting clearance, energetic support and plasticity; they also modulate the blood–brain barrier and brain immune response. Therefore, these cells integrate different signals—metabolic, immune and neural inputs. Astrogliosis refers to astrocyte reactivity in all forms of injury and the increment of glial fibrillary acidic protein (GFAP) is its most frequent marker [7]. However, GFAP up-regulation on its own provides little information concerning reactive astrocyte function [8]. From our experience, changes in extracellular levels of S100beta have been observed in acute and chronic neuroinflammatory conditions, associated with neurodegenerative processes, like those occurring in Alzheimer´s disease and other systemic diseases displaying neuropsychiatric disorders [2,9]. This lecture highlights how during injury, astrocytes are simultaneously exposed to a myriad of stimuli from microglia, blood and neurons, leading to a complex reactivity whose signaling will result, or not, in a dysfunctional cell, Figure 1. At the same time, it is necessary to investigate other specific astrocyte markers, such as aquaporin-4, glutamate transporters and S100beta, as well as the transcription factors involved. Finally, it was important to consider that potential targets to treat neuroinflammation such as immune receptors (e.g., TNFR2: tumor necrosis factor repceptor 2), protein phosphatases (e.g., calcineurin) and protein kinases (e.g., p38) also are actively present in astrocytes [10,11].

Guanosine Neuroinflammation and Neuroprotection

Roberto Farina de Almeida, Elaine Elisabetsky and Diogo Onofre Gomes Souza. E mail: almeida_rf@yahoo.com.br 

**Figure 2:**
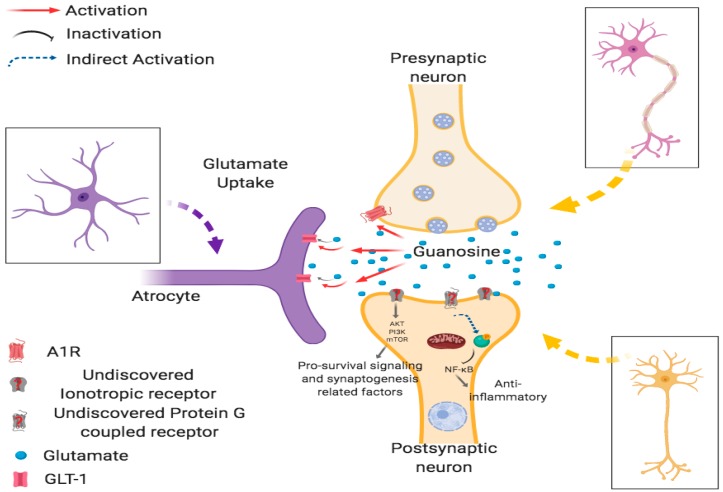
Postulated pathways involved in GUO mechanism of action. Figure was produced with permission using Biorender free version (www.biorender.co). A1R: Adenosine a1 receptor, GLT-1: Glutamate transporter-1

Inflammation in the central nervous system (CNS) plays an important role in several brain disorders [12]. Glutamate, an excitatory neurotransmitter essential for most brain activities, has been considered relevant to the pathogenesis of neuroinflammation, contributing to the development of several acute and chronic brain injuries [13]. The main process responsible for maintaining extracellular glutamate levels below toxic concentrations, thus favoring the physiological glutamatergic tonus, is the glutamate uptake activity of glutamate transporters located in neural cell membranes, mainly in astrocytes [14]. In this context, our group presented strong evidence that systemic guanosine (GUO) administration is effectively neuroprotective against glutamate toxicity and neuroinflammation in different protocols that mimic several brain disorders, in both in vitro and in vivo studies, Figure 2 [15]. These results point to a potential applicability of the neuroprotective effects of GUO in putative translational studies [16]. In this topic, the relevance of these data were discussed, accompanied by our more recent results regarding the potential antidepressive-like effect exerted by acute and chronic GUO treatment in mice submitted to a well-validated animal model of depression. In addition, we also demonstrated that GUO treatment attenuated hippocampal redox imbalance, accompanied by a significant modulation in peripheral and central (hippocampal) inflammatory cytokines. In this line of research, our results establish new perspectives for therapeutic developments regarding the latest results related with the antidepressive-like effects of acute and chronic GUO in mice subjected to olfactory bulbectomy, a well-validated rodent model of depression with translational value. In addition to the behavioral data, we show that GUO attenuates hippocampal redox imbalance and significantly modulates peripheral and central (hippocampal) inflammatory cytokines.

Neuroinflammation in Brain Ischemia 

Alina González-Quevedo, Marisol Peña Sánchez, María Caridad Menéndez Saínz, Rebeca Fernández Carriera, Anay Cordero Eiriz, Melany Betacourt Loza. E mail: aglez@infomed.sld.cu

According to the global burden of stroke study conducted in 2013, stroke is the second most common cause of death worldwide (11.8%), preceded only by ischemic heart disease (14.8%), and it constitutes the major cause of severe neurological deficits in the adult population, ischemic stroke accounting for ~85% of all strokes [17]. Several intricately interrelated mechanisms are known to participate in the development of the ischemic lesion, such as excitotoxicity, inflammation, blood–brain barrier disruption, complement cascade activation and increased free radical release. Nevertheless, inflammatory signaling is present throughout the ischemic cascade, from the early acute ischemic injury that follows arterial occlusion to the final regenerative processes which underlie post-ischemic tissue repair [18].

**Figure 3:**
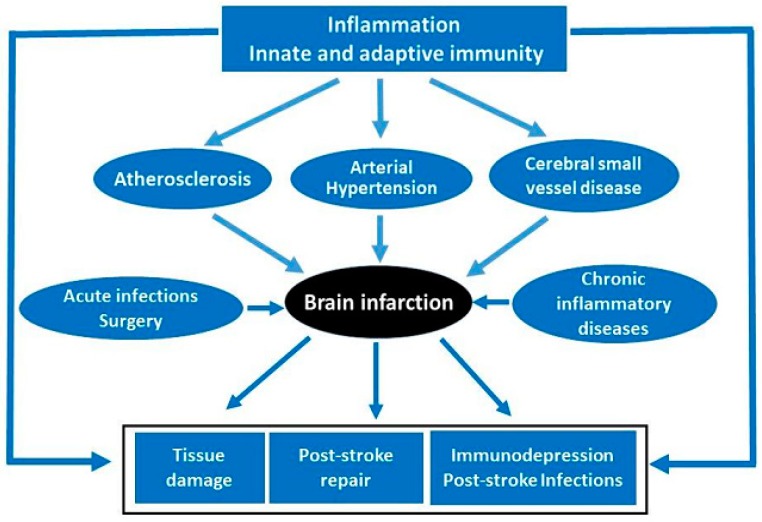
Inflammation and immunity events preceding and following acute ischemic stroke.

Inflammatory and immune components also prevail in conditions preluding the acute ischemic event: atherosclerosis, the basis of large and medium-sized artery disease, cerebral small vessel disease and hypertension—the main risk factor for cerebrovascular diseases (twice as important as for coronary heart disease). Activation of the immune system is thought to increase the risk of stroke. This is mainly based on the association observed between stroke and antecedent acute and chronic inflammatory states. Acute brain ischemia also exerts a potent suppressive effect on lymphoid organs, predisposing the patients to concomitant infections, thus complicating the outcome of stroke in terms of morbidity and mortality [18,19] Figure 3. 

In this approach, we will engage in the involvement of inflammation and immunity in the medical conditions that antecede acute ischemic stroke, as well as highlight how these processes are involved in the pathophysiology of the ischemic lesion once established and in the subsequent repair mechanisms [4,20].

Novel Treatment Neuroimmunomodulators in Multiple Sclerosis Relapsed Remission

Sandra Chow and Amado Díaz de la Fé.E mail: sandra.chow@merckgroup.com

**Figure 4:**
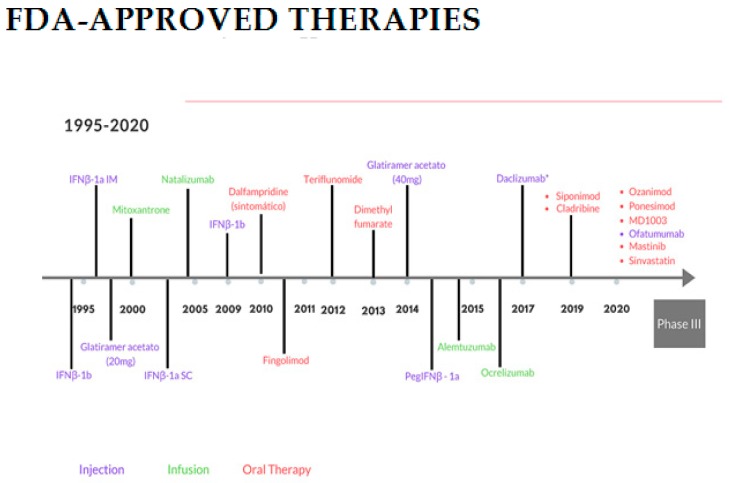
The Evolving multiple sclerosis (MS) Treatment Landscape.

Multiple sclerosis is the most common potential cause of neurological disability in young adults without history of trauma. The disease has two different clinical phases, which reflect a dominant state of the pathological processes: (1) inflammation corresponding to activity during the relapsing-remitting stage and (2) axon degeneration representing the main substrate of progressive disability. Most of the traditional disease-modifying drugs are involved in the inflammatory process. In 2019, three medications were approved for the progressive form of multiple sclerosis (siponimod, ocrelizumab and cladribine). Therefore, the question is to develop strategies that promote remyelination and prevent axonal loss. Currently, the pharmacological contribution of the therapeutic arsenal for multiple sclerosis (MS) treatment during the last 20 years has been focused on targeting the inflammatory process in the CNS. Both physicians and patients are demanding therapies, focused treatment with high efficiency, short administration, simple monitoring and a high safety profile. Figure 4 shows the disease modifying drugs in the market in recent years [21], however there are still unmet needs for both the clinician and the patient. It is important to be aware of making a good diagnosis and selecting the ideal medication for the patient at the right time.

Disease-modifying therapy in MS is a key component of comprehensive MS care, along with managing MS relapses, treating symptoms and paying attention to overall health and wellness. Disease modifying medications are, at this time, the most valuable strategy available to slow the natural course of the disease, Figure 4 [22]. Even though these medications do not generally make the patient feel better, they can be looked upon as an investment for the future. Clinical studies have demonstrated that all of the medications for relapsing forms of MS reduce the frequency and severity of clinical attacks by 28–68%, reduce the development of new damaged areas in the brain and spinal cord as seen on MRI (magnetic resonance imaging), toward fewer, smaller or no new lesions on MRI scans and slow the accumulation of disability to delay progression. 

Subsequent research and clinical experience indicate that early treatment with these disease- modifying drugs may help to prevent permanent damage in the CNS. Permanent damage to axons occurs in MS. Overall brain atrophy shrinkage can occur early in the disease, and damage can continue even when a person has no symptoms and feels well. At the same time, it must be taken into account that the FDA (Food and Drug Administration) for pregnant women, or those who are breastfeeding, have approved none of these medications. 

In the last two decades, 15 medications have been proposed as modifiers of the disease for MS, being evaluated not only in terms of effectiveness, but also regarding their validity for intervention monitoring, treatment adherence, incidence of co-morbidities and secondary effects, among other aspects. This conference offered an updated vision to personalize the ideal medication for the control of MS through the combination of these new therapies recently approved for RRMS (Relapsing Remmiting Multiple Sclerosis).

Biomarkers and Prodromal Stage in Spinocerebellar Ataxia Type 2 

Luis Velázquez-Pérez. E mail: velazq63@gmail.com 

Spinocerebellar Ataxias (SCAs) are a genetically heterogeneous group of autosomal dominant neurodegenerative disorders. The global prevalence is about 3 cases/100,000 inhabitants and the age at onset is usually between 2 and 50 years of age. Unfortunately, there is no treatment available for these disorders. SCA3 is the most common SCA worldwide, followed by SCA2 and SCA6. Spinocerebellar ataxia type 2 (SCA2) is caused by the abnormal expansion of Cytosine-Adenine-Guanine (CAG) triplet repeats in the coding region of the ataxin-2 gene (12q24.1). The epidemiological studies have demonstrated that the highest prevalence rates of SCA2 patients and SCA2 mutations are in Holguin province, Cuba. The nationwide frequency of SCA2 in Cuba is around 85%. 

All patients show a progressive cerebellar syndrome characterized by gait ataxia, incoordination of the upper and lower limbs and cerebellar dysarthria. They also display slowing of saccadic eye movements, peripheral neuropathy, signs of motor neuron involvement such as fasciculations and amyotrophy, autonomic abnormalities (urinary dysfunction, hypohydrosis and constipation), sleep disturbances (restless legs syndrome, muscle cramps and insomnia) and cognitive disorders (frontal-executive dysfunctions as well verbal memory deficits) [23]. 

The identification of biomarkers is very important because they allow the selection of patients for clinical trials, improving clinical diagnosis, staging of the disease, evaluation of the genetic damage, providing the physiopathological clues and assessing efficacy of clinical trials. They can be classified as neurophysiological, quantitative paraclinical, imaging and biochemical biomarkers. These objective parameters can provide quantitative evidence to classify SCA2 into distinct disease stages considering the presence of motor and nonmotor features. 

**Figure 5:**
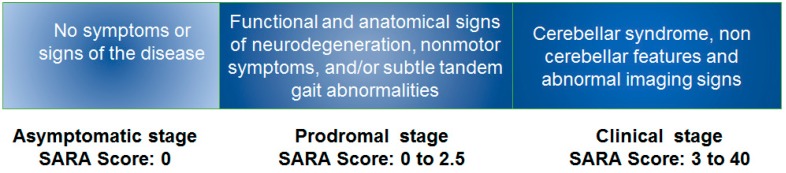
Natural history of the progression of the SCA2 cerebellar ataxia. SARA: Scale for the Assessment and Rating of Ataxia.

The natural history of the progression of SCA2 can be classified according to the phenotypical features in three stages: asymptomatic, prodromal and clinical or ataxic stages (Figure 5) [21]. To characterize these phases, two important studies were done: a cross-sectional evaluation and a longitudinal follow-up study in SCA2 preclinical subjects. In the asymptomatic stage, no disease symptoms or signs nor electrophysiological abnormalities and imaging signs are detectable. This phase is followed by the prodromal or preclinical stage, characterized by unspecific neurological features such as painful muscle cramps, sensory abnormalities, subtle cerebellar manifestation (increase of the sway during tandem gait), cognitive decline, autonomic disorders, olfactory dysfunction and hyperreflexia. Electrophysiological studies showed the reduction of the maximal saccadic velocity, REM sleep disorders, increased central motor conduction time in transcranial magnetic stimulation and peripheral sensory axonal damage according of the peripheral nerve conduction studies [3,24]. 

The prodromal stage in spinocerebellar ataxia type 2 provides insight into the physiopathology of neurodegeneration before cerebellar syndrome onset, allowing the design of clinical trials before ataxia onset—when neurodegeneration is still incipient—to slow down disease progression. This stage also allows identification of the best moment to initiate therapies, and identification of sensitive outcome measures [24]. 

## 3. Conclusions

Inflammatory processes have been established as major components in the pathophysiology of neurological diseases, including genetic and non-genetic disorders, which display behavioral impairment. Based on this knowledge, this meeting was focused toward the discussion of neuroimmune mechanisms and neuromodulatory therapeutic approaches related to brain diseases and tools for their modification. In conclusion, the meeting emphasized the main role of neuroinflammation in the pathophysiology of these disorders, as well as neuromodulation as an interventional tool to modify or control disease course and progression.
